# Tumour markers of prognosis in colorectal cancer

**DOI:** 10.1038/sj.bjc.6690033

**Published:** 1999-01

**Authors:** H L McLeod, G I Murray

**Affiliations:** Department of Medicine & Therapeutics, University of Aberdeen, Foresterhill, Aberdeen, AB25 2ZD, UK; Department of Pathology, University of Aberdeen, Foresterhill, Aberdeen, AB25 2ZD, UK

**Keywords:** colorectal cancer, prognosis, immunohistochemistry, tumour marker


*British Journal of Cancer* (1999) **79**(2), 191–203
© 1999 Cancer Research Campaign

Review

### Tumour markers of prognosis in colorectal cancer

**HL McLeod[1] and GI Murray[2]**

Departments of [1]Medicine & Therapeutics, and [2]Pathology, University of Aberdeen, Foresterhill, Aberdeen AB25 2ZD, UK

**Keywords:** colorectal cancer; prognosis; immunohistochemistry; tumour markers

Colorectal cancer is a common disease in developed nations. In Europe, there are approximately 210 000 new cases per year and around 124 500 people die from this disease each year (Parkin et al, 1993). Thus, it is the second most common malignancy in men and third most common in women. About one-third of patients have a primary tumour in the rectum with the majority of the remaining tumours occurring in the sigmoid colon. Patients who die from the disease most commonly succumb to the effects of secondary metastasis to the liver. The prognosis for this cancer is influenced by a variety of features present at the time of initial diagnosis. These factors include age, gender, duration of symptoms, presence of bowel obstruction, tumour location, need for blood transfusion and the quality of surgical intervention (Wolmark et al, 1983; McArdle and Hole 1991; Hermanek et al, 1995). A number of tumour characteristics, such as vascular and lymphatic invasion, ploidy, differentiation and preoperative levels of plasma carcinoembryonic antigen, have also been shown to be of prognostic significance (Witzig et al, 1991; Hermanek et al, 1995). However, the simplest and most widely applied prognostic feature is the presence of lymph node metastasis in a surgical resection specimen. This forms the basis of all staging systems for this disease and has repeatedly been shown to have more prognostic power than any other single feature at presentation (Astler and Coller, 1954; Cohen et al, 1991).

Treatment of colorectal cancer is unsuccessful if the tumours have an aggressive phenotype at the time of diagnosis. To date, the most important variable influencing the long-term outcome from colorectal cancer is the stage of disease at diagnosis, with depth of tumour invasion and infiltration of lymph nodes or distant organs being used to define patient prognosis (Table 1). This classification discriminates patients with early-stage disease from those with very advanced disease, but is less able to predict the prognosis of patients with intermediate levels of tumour invasion. For example, in the Intergroup study of 929 patients with Dukes' C colorectal cancer, 45% of patients treated with surgery alone were alive and disease free after 5 years compared with 65% of patients treated with surgery followed by 5-fluorouracil(5-FU)/levamisole (Moertel et al, 1995). This suggests that, in retrospect, nearly half of the patients with Dukes' C colorectal cancer did not require adjuvant chemotherapy and 35% will die even when treated with systemic chemotherapy. These data highlight the need for informative prognostic markers which will aid identification of patients best treated with surgery alone, those requiring chemotherapy and those who might benefit from more aggressive or experimental therapy.

*Received 20 March 1998*
*Revised 1 June 1998*
*Accepted 4 June 1998*

*Correspondence to:* HL McLeod

### DEFINITION OF PROGNOSTIC MARKER

A practical definition of a prognostic marker is a variable that provides prospective information on patient outcome which is complementary to the data obtained by the pathologist from the diagnostic sections and on which therapeutic decisions can be guided. As Dukes' staging is widespread and common practice, there would be no logic in finding a marker that simply gave the same information (Astler and Coller, 1954; Cohen et al, 1991). Rather, proteins (or other variables) which demonstrate a strong correlation with Dukes' stage (or other staging systems) should be considered as possible novel targets for anti-cancer therapies. Dukes' stage does provide a useful 'internal control', as it is the gold standard on which prognostic markers should be compared, and any study including Dukes' A to D disease that does not demonstrate a relationship between stage and survival should be viewed with great suspicion.

A large number of prognostic markers for colorectal cancer are featured in the medical literature. Almost all studies of prognostic markers in colorectal cancer involve the correlation of variables observed at diagnosis with eventual outcome. Most published studies have used a single marker, while a minority of studies have used a small group of prognostic markers from which clinical correlations were derived. Although there are many claims of prognostic significance in colorectal cancer the question remains: which markers are important enough that they should be applied prospectively in routine pathological evaluation of colorectal cancer?

### INCLUSION OF TREATMENT DATA

One difficulty with many prognostic marker studies is their applicability to modern practice. A study demonstrating that overexpression of marker 'X' gives a survival advantage in patients with Dukes' stage C disease may have little relevance if the data are derived from a population treated prior to the routine use of adjuvant chemotherapy. This puts constraints on the length of clinical follow-up information available, as chemotherapy only became standard practice within the last 5–7 years in most centres. The details of patient therapy are often inadequate in published studies

**Table 1** Comparison of the two most commonly used pathological staging systems for colorectal cancer

| | Dukes' stage | AJCC/UICC stage |
|---|---|---|
| Tumour invading the submucosa or muscularis propria | A | I |
| Tumour invading through the muscularis propria | B | II |
| Evidence of tumour in regional lymph nodes, regardless of local tumour invasion | C | III |
| Distant metastasis, regardless of local tumour invasion or lymph node status | D[a] | IV |

[a]Stage D was not included in the original Dukes' staging system, but has become commonly used to represent distant metastasis.

of prognostic markers. It is not satisfactory for the degree of heterogeneity of treatment regimens within a study to be greater than the degree of tumour heterogeneity for the marker under evaluation.

### CHOICE OF STUDY POPULATION

The choice of study population needs to be carefully considered for studies of markers of potential prognostic importance. Many published studies include patients with all Dukes' stages (A to D), which is useful for initial evaluation of putative markers of prognosis. However, subsequent studies of patients with a specific disease stage are then more appropriate for the assessment of the clinical utility of a marker. For example, a study of 200 patients with Dukes' stage C disease will have greater statistical power to yield definitive results than a study which consists of 20 Dukes' A, 80 Dukes' B, 80 Dukes' C and 20 Dukes' D patients. In addition to diluting the statistical power of the study, the inclusion of all stages of disease is inappropriate for the question asked, as there is little potential for the clinical use of markers in stage A disease and markers of tumour response to therapy or quality of life are more applicable to stage D patients. The time scale in which the samples are collected also has a significant influence on the applicability of the results to modern cancer treatment. As mentioned above, the treatment of colorectal cancer has changed significantly over the past 10 years, and this changes the utility of prognostic findings. Specialization of both surgeons and oncologists has improved patient outcome (Hoffman et al, 1997). The technical ability of the surgeon is also a relevant variable which should not be ignored in studies of prognosis (Reinbach et al, 1994). The processing of tumour specimens can alter the application of many techniques. For example, the type of formalin and length of time in fixative can alter the protein structure of target molecules, affecting the use of many antibodies. These factors can also alter the integrity of genomic DNA extracted from the tissue sections and thereby interfere with microsatellite analysis or other DNA-based techniques. The development of national guidelines for the processing of tumour samples (such as the *UKCCCR Handbook for the Clinico–Pathological Assessment and Staging of Colorectal Cancer*) should lead to a more uniform handling of sample material.

### THE ENRICHMENT APPROACH

It is not practicable to test all potential markers of prognosis in a large data set of tumours of a particular Dukes' stage. One potential strategy for providing initial data for putative prognostic marker studies is the use of an 'enrichment' approach. With this approach, a data set of patients could be retrospectively selected based on their clinical phenotype, such as 5-year survival or response to chemotherapy. By selecting patients at the extremes of the desired endpoint (i.e. Dukes' C patients alive 5 years post adjuvant therapy vs patients who developed metastatic disease while receiving adjuvant therapy) an 'enrichment' step should take place. Variables only important for tumorigenesis should be equally distributed between the two patient groups, while markers of tumour invasion, chemosensitivity or other factors regulating the efficacy of adjuvant therapy should be delineated between the patient groups. This approach assumes a minimum degree of heterogeneity in therapeutic regimens and can only be performed using retrospective analysis. It also relies on the availability of the relevant pathological specimens, often limiting laboratory approaches to those that are applicable to formalin-fixed tissues. There are, however, statistical concerns about the use of a large number of potential tumour markers in what will often be a small number of clinical specimens. However, the goal is to identify markers with a clear association with outcome, and clinical significance needs to have a greater role than statistical significance. A protein that is expressed in 30% of survivors and 0% of patients with disease progression will be taken forward to more definitive analysis, regardless of the statistics. This approach will not prove the clinical utility of a marker, but rather should be a fertile ground for identifying putative markers for further analysis in large studies of specific tumour stage.

In this review, the current data on cellular or molecular markers with the potential for prognostic predictive ability for patients with colorectal cancer are summarized. It would not be possible to explore all of the putative prognostic markers in the literature, therefore those for which literature is available from either large single studies or analysis from multiple laboratories were selected.

**Table 2** Comparison and applicability of different methodologies for assessment of tumour markers

| | PCR | PCR SSCP | LOH | RT-PCR | Northern blotting | ISH | IHC |
|---|---|---|---|---|---|---|---|
| Cellular constituent examined | DNA | DNA | DNA | mRNA | mRNA | DNA/mRNA | Protein |
| Use in formalin-fixed, wax-embedded tissue | Y | Y | Y | N | N | Y | Y |
| Microdissection needed | Y[a] | Y[a] | Y[a] | Y[a] | Y[a] | N | N |
| Cellular localization evaluable | N | N | N | N | N | Y | Y |
| Application to routine diagnosis | Y[b] | N | N | N | N | Y[b] | Y |

IHC, immunohistochemistry; ISH, *in situ* hybridization; LOH, loss of hetorozygosity; PCR, polymerase chain reaction; RT, reverse transcriptase, SSCP, single-strand conformational polymorphism. [a]Depending on the cellularity of colorectal tumours, microdissection may be desirable to ensure an enriched population of tumour cells. [b]Academic centres and larger general hospitals.

 © Cancer Research Campaign 1999

Papers were identified from the Institute for Scientific Information database (through the Bath Information and Data Services) using the search terms colon, rectal or colorectal with prognosis, prognostic or survival. The search was conducted from January 1990 to January 1998 and was restricted to English-language publications. Negative studies may be under-represented in the literature because of the personal and editorial bias against publishing such work. The markers can be separated into oncogenes, tumour-suppressor genes, proteins involved in apoptosis, factors involved in angiogenesis/metastasis/invasion and cellular targets for the chemotherapy used for colorectal cancer. An emphasis has been placed on techniques that use immunohistochemistry, thereby potentially enabling the results to be applied in many different settings throughout the world. However, a spectrum of methodologies are available at most medical centres to assess tumour markers (Table 2).

### ONCOGENES

### Ras

The *ras* family represent one of the most commonly detected oncogenes in human cancer. The *ras* genes (K-*ras*, H-*ras*, N-*ras*) encode for 21-kDa proteins (p21s), which are localized on the inner surface of the plasma membrane. These proteins bind guanine nucleotides and have GTPase activity. As with other GTP-binding proteins, ras may serve as a transducer molecule for signals affecting cell proliferation and thus have involvement in the cell cycle. The development of *ras* mutations is thought to be an early event in the current model of colorectal tumorigenesis (Fearon and Vogelstein, 1990). The prognostic significance of both *ras* mutations and p21 staining has been assessed in a number of reports, with conflicting results.

The majority of *ras* mutations are found in codon 12, with additional mutations observed in codons 13 and 61. Nested polymerase chain reaction (PCR) methods for detection of *ras* mutations in DNA isolated from formalin-fixed, paraffin-embedded colorectal tumour have allowed assessment of prognostic significance using retrospective analysis of large numbers of patients. Although there are data in the literature on *ras* mutations and survival from over 1200 patients, the clinical relevance of these alterations is not yet clear. At least four studies with a total of 498 patients demonstrate that the presence of a *ras* mutation at either codon 12 or 13 is associated with a poor prognosis, independent of Dukes' stage (Benhattar et al, 1993; Elnatan et al, 1996; Smith et al, 1996*a*; Span et al, 1996). In addition, two studies with a total of 227 patients found a poorer prognosis in patients with mutations in both *ras* and *p53*, even when either alteration alone was not an independent predictor of survival (Bell et al, 1993; Smith et al, 1996*a*). A single study found that *ras* mutations were associated with tumour progression and poor survival in patients with left-sided tumours, but not in patients with right-sided disease (Elnatan et al, 1996). There was no substantial difference in study design or patient selection between the studies listed above and the seven studies (a total of 610 patients) in which the presence of *ras* mutations was not associated with prognosis (Dix et al, 1994; Bennett et al, 1995; Markowitz et al, 1995; Elnatan et al, 1996; Pricolo et al, 1996; Andersen et al, 1997; Wadler et al, 1997). Most of the studies in which no significance was identified had restricted analysis to a specific pathological stage of disease [Dukes' B (Bennett et al, 1995); Dukes' B/C (Dix et al, 1994); Stage III (Pricolo et al, 1996); advanced disease (Wadler et al, 1997); Markowitz et al, 1995], while the studies demonstrating a positive correlation with prognosis included a mix of Dukes' A–D tumours. It is possible that the presence of *ras* mutations is associated with disease invasiveness, independent of pathological stage, but is not of prognostic significance within a given stage of disease. This may account, in part, for an association with survival in the studies including Dukes' A–D tumours and the lack of prognostic importance in the studies including larger numbers of patients with a specific pathological stage.

The influence of *ras* mutations on response to 5-FU-based chemotherapy has been evaluated in 108 patients (Markowitz et al, 1995; Wadler et al, 1997). An objective response rate of 39% was achieved with the different regimens, but mutant *ras* was not associated with chemosensitivity.

Immunohistochemical analysis of ras p21 staining and outcome has been performed in four studies (a total of 399 patients) (Miyahara et al, 1991; Sun et al, 1991; Miller et al, 1992; Bennett et al, 1995). Different monoclonal antibodies were used in all studies and the method of scoring a tumour p21 as 'positive' either was not detailed or differed between the studies. Tumour staining for p21 was found in 54% (216/399) of tumours, but varied between studies (31–71% positive). Positive staining for p21 was associated with a poor prognosis in three out of four studies (Miller et al, 1992; Sun et al, 1991; Miyahara et al, 1991) and was shown to be an independent prognostic factor in only one study (Sun et al, 1991). Although there are concerns about the methodology (non-specific ras antibody, criteria for 'positive' expression) and statistical analyses used in these studies, there is an overall indication that tumour staining for p21 is associated with a poor prognosis and should be considered for inclusion in more definitive studies of markers of prognostic significance in colorectal cancer.

### Transforming growth factor (TGF) α and β-1

The transforming growth factor (TGF) superfamily consists of several subgroups, including α and β, of which there are a number of isoforms (Kumar et al, 1995; Lawrence, 1996). TGF-α produces a mitogenic effect through interaction with the epidermal growth factor receptor and promotes the growth of colon cancer cells in vitro in an autocrine manner. The role of TGF-α as a marker of survival in colorectal cancer was evaluated in 105 patients (stage not given) who were treated with surgery alone (Younes et al, 1996). Patients with low TGF-α expression (<25% positive cells) had a poorer prognosis than those with >25% positive staining ($P$ = 0.03). Although this brief report did not evaluate the independence of TGF-α as a prognostic marker, the significant difference in overall survival makes it a candidate for further evaluation.

TGF-β-1 is the most abundant member of the TGF-β family. TGF-β-1 inhibits the growth of epithelial cells but stimulates mesenchymal cell proliferation and cell migration. Resistance to TGF-β-1-induced growth inhibition is observed in colorectal cancer and is associated with tumour progression in laboratory models (Robson et al, 1996). TGF-β-1 staining was evaluated in formalin-fixed tissue from 72 patients with colorectal cancer (Dukes' stage A, 5; B, 33; C, 16; D, 18) (Robson et al, 1996). Positive expression was observed in 58% of tumours and was associated with Dukes' classification (Dukes' D, 72% vs Dukes' A, 40%; $P$ < 0.05). Poorer survival was observed in patients with positive staining for TGF-β-1. This was more apparent for patients receiving a palliative

© Cancer Research Campaign 1999

resection of their primary tumour than for patients receiving a curative resection (Robson et al, 1996). Unfortunately, evaluation of the independence of TGF-β-1 staining from Dukes' stage was not reported in this study. However, the data suggest a role for TGF-β-1 staining in the context of advanced disease.

### *c-erb*B2

The *c-erb*B2 proto-oncogene (also called *Her*-2, *neu*) is a 185-kDa glycoprotein with tyrosine kinase activity. Expression of *c-erb*B2 has demonstrated prognostic significance in breast and gastric cancer. However, it has not been extensively evaluated in colorectal tumours. A study of 151 adenocarcinomas (Dukes' stage A, 34; B, 40; C, 77) identified a significant relationship between *c-erb*B2 immunohistochemistry and patient survival (Kapitanovic et al, 1997). *c-erb*B2 expression was found in all cases and was strongly positive in 43% of tumours. An inverse correlation between *c-erb*B2 staining and survival was noted, with median survival of 120 weeks and 28 weeks for tumours with moderate and strong staining for *c-erb*B2 respectively. Median survival for patients with weak staining was greater than 275 weeks. Although *c-erb*B2 expression was significantly correlated with Dukes' stage, *c-erb*B2 was an independent marker of prognosis in this study. *c-erb*B2 staining was also of prognostic importance in a study of 164 Dukes' B tumours (Kay et al, 1994). Cytoplasmic staining was observed in 33.5% of tumours and was a poor prognostic indicator (5-year survival 47% compared with 77% in *c-erb*B2-negative tumours). *c-erb*B2 staining was also an independent prognostic marker in 293 tumours (stage not stated) (Sun et al, 1995). However, unlike the previous studies, a favourable prognosis with overexpression was observed. This finding was primarily confined to aneuploid tumours. This discrepancy in the prognostic significance of *c-erb*B2 overexpression needs to be more definitively addressed before this marker can be recommended for use in the management of patients with colorectal cancer.

### Epidermal growth factor receptor

Epidermal growth factor receptor (EGFR) is a 170-kDa transmembrane protein with tyrosine kinase activity. The receptor is able to bind epidermal growth factor and to transduce the signal into the cytoplasm. Expression of EGFR is of prognostic significance for a number of solid tumours, including breast and ovarian carcinoma. EGFR was evaluated in 82 patients with colorectal cancer (Dukes' stage A, 4; B, 41; C, 22; D, 15; Mayer et al, 1993). Patients with Dukes' C disease received adjuvant 5-FU and levamisole, while Dukes' D patients received 5-FU and leucovorin. EGFR-positive staining was detected in 80/82 (97.6%) colorectal tumours, with the majority (62.2%) having >50% positive cells. Patients with >50% positively staining cells had a poorer survival (median 22.5 months) than those with a lower degree of expression (>60 months; $P < 0.01$) (Mayer et al, 1993). The role of EGFR as an independent prognostic marker was not clearly defined, but Dukes' stage and lymph vessel invasion were also shown to be of prognostic importance. A second study evaluated EGFR in 84 patients with Dukes' B colorectal carcinomas who had 10 years of follow-up information (Linden et al, 1996). Although 52% of tumours expressed EGFR, no association was found between EGFR and survival (Linden et al, 1996). These two studies leave a great deal of uncertainty regarding the role of EGFR in colorectal cancer. There is a clear need for a further large study, preferably in patients with Dukes' B and C disease, to define the utility of EGFR immunohistochemistry as a marker of prognosis in colorectal cancer.

### c-*myc*

Amplification of the cellular oncogene c-*myc* has been described in both colorectal cell lines and primary tumours. c-*myc* gene copy number was determined using a dot-blot technique in tumour DNA from 149 patients with Dukes' B or C colon cancer treated on Intergroup study EST2284 (surgery vs surgery + levamisole vs surgery + 5-FU + levamisole) (Augenlicht et al, 1997). Using an arbitrary cut-off point of 1.5 to define amplification, 32% of patients had elevated c-*myc*. Among patients with no c-*myc* amplification there was no difference in survival between the three treatment arms. However, c-*myc*-positive patients treated with surgery + 5-FU + levamisole ($n = 12$) had a longer time to disease progression ($P < 0.05$) and a trend towards longer survival ($P = 0.09$) than c-*myc*-positive patients treated with surgery alone ($n = 25$) or surgery + levamisole ($n = 11$). While the small number of patients with c-*myc*-positive tumours in each treatment group and the failure to assess the independence of c-*myc* as a marker of prognosis limit the interpretation of this study, it does suggest a role for c-*myc* detection in the selection of patients with Dukes' stage B or C colon cancer for whom adjuvant chemotherapy is indicated.

RNA-based analysis of the prognostic impact of c-*myc* expression has also been performed in 119 patients (Dukes' stage A, 31; B, 27; C, 29; D, 32) and 29 patients (Dukes' stage A, 2; B, 10; C, 10; D, 7) (Nagai et al, 1992; Smith and Goh, 1996). In addition, analysis of c-*myc* DNA copy number was performed in the smaller study (Nagai et al, 1992). Neither c-*myc* gene copy number nor RNA expression was of prognostic significance in the smaller study (Nagai et al, 1992). In contrast, c-*myc* overexpression was observed in 60% of cases in the larger study and these patients had a better prognosis than patients without overexpression ($P = 0.02$) (Smith and Goh, 1996). However, the authors found that the beneficial effect of c-*myc* overexpression was restricted to those tumours containing wild-type *p53* (Smith and Goh, 1996). This highlights the fact that evaluation of a specific prognostic marker cannot be carried out in isolation but needs to be studied in the context of a more complex, multigenic environment.

Immunohistochemical analysis of c-myc protein in colorectal tumours has been evaluated in two separate studies [$n = 118$, Dukes' A, 7; B, 35; C, 41; D, 35 (Miller et al, 1992) and $n = 48$, Dukes' B, 24; C, 24 (Bhatavdekar et al, 1997)]. Positive staining for c-myc was found in 71% and 65% of patients, respectively, and was not associated with Dukes' stage or histological grade in either study (Miller et al, 1992; Bhatavdekar et al, 1997). A significantly better overall survival was observed in the 17 patients without c-myc staining, when compared with the 31 with positive staining in the smaller study (Bhatavdekar et al, 1997). However, c-myc was not of prognostic importance in the larger study (Miller et al, 1992).

### TUMOUR-SUPPRESSOR GENES

### p53

The p53 tumour-suppressor gene is a commonly altered gene in human solid tumours. The gene has been localized to chromosome 17p13.1, a region of allelic deletion in many tumours. The remaining

 © Cancer Research Campaign 1999

**Table 3** Molecular and cellular evaluation of p53 as a marker of prognosis in colorectal cancer. All immunohistochemistry (IHC) studies were performed on formalin-fixed sections and the antibody used is listed in parenthesis. Studies evaluated nuclear staining for p53 unless otherwise indicated. The studies presented here were selected from the literature based on large sample number or focus on a specific tumour stage

| Technique | Number of tumours | Significance | Reference |
|---|---|---|---|
| *Immunohistochemical analysis* | | | |
| IHC (DO-1) | Dukes' A = 42<br>Dukes' B = 82<br>Dukes' C = 84<br>Dukes' D = 45 | Overexpression associated with poor survival in Dukes' B, but not Dukes' A, C, D | Bartik et al (1997) |
| IHC (DO-7) | Dukes' A = 54<br>Dukes' B = 133<br>Dukes' C = 73<br>Dukes' D = 34 | Not an independent predictor of survival | Kressner et al (1996) |
| IHC (CM-1) | Dukes' A = 43<br>Dukes' B = 101<br>Dukes' C = 94<br>Dukes' D = 50 | Cytoplasmic, but not nuclear, overexpression = poor survival<br>Independent | Sun et al (1992) |
| IHC on endoscopic biopsies (1801) | Dukes' A = 25<br>Dukes' B = 68<br>Dukes' C = 55<br>Dukes' D = 55 | Overexpression = poor prognosis<br>Independence not assessed | Yamaguchi et al (1993) |
| IHC (CM1) | Stage I = 35<br>Stage II = 78<br>Stage III = 70<br>Stage IV = 23 | Cytoplasmic, but not nuclear, overexpression = poor survival<br>Independent factor | Bosari et al (1994) |
| IHC (DO-7) | Stage I = 9<br>Stage II = 80<br>Stage III = 69<br>Stage IV = 46 | Not an independent predictor of survival | Lanza et al (1996) |
| IHC (DO-7) and PCR–SSCP exons 5–8 | Dukes' A = 14<br>Dukes' B = 21<br>Dukes' C = 31 | Overexpression = poor survival<br>Independent factor<br>Mutation only important for Dukes' A and B ($P = 0.01$) | Leahy et al (1996) |
| IHC (DO-1) | Dukes' A = 29<br>Dukes' B = 88<br>Dukes' C = 130 | Not an independent predictor of survival | Poller et al (1997) |
| IHC (DO-7) and PCR + SSCP exons 5–8 | Dukes' B = 263<br>Dukes' C = 278 | Overexpression = good prognosis, most significant for distal tumours<br>Independent factor | Soong et al (1997) |
| IHC (DO-1) | Stage IB = 53<br>Stage II = 48 | Overexpression = poor survival<br>Independent factor | Baretton et al (1996*a*) |
| PCR-RFLP exon 4 plus IHC (CM1) | Dukes' B = 168 | Not associated with survival | Bennett et al (1995) |
| IHC (1801) | Dukes' C = 107 | Overexpression = poor survival<br>Cytoplasmic p53 not associated with survival | Zeng et al (1994) |
| IHC (1801) | Dukes' D = 50, all treated with intrahepatic chemotherapy | Overexpression = poor survival | Belluco et al (1996) |
| IHC (DO-1) | Dukes' D = 59 | Overexpression associated with lower response ($P < 0.03$), but not survival | Brett et al (1996) |
| IHC (1801) | Dukes' D = 59 | Overexpression not associated with relapse or survival | Costa et al (1997) |
| IHC (1801) | Dukes' D = 71 treated with 5-FU modulated with methotrexate | Not associated with response to therapy or survival | Paradiso et al (1996) |

© Cancer Research Campaign 1999

**Table 3** Cont

| Technique | Number of tumours | Significance | Reference |
|---|---|---|---|
| *Molecular analysis* | | | |
| RT-PCR-SSCP | Dukes' A–D, *n* = 192 | Mutation = poor survival Independent of stage (*P* = 0.031) | Goh et al (1995) |
| IHC (DO-7) and PCR–SSCP exons 5–8 | Dukes' A = 14 Dukes' B = 21 Dukes' C = 31 | Mutation = poor survival for Dukes' A and B | Leahy et al (1996) |
| PCR–RFLP exon 4 plus IHC (CM1) | Dukes' B = 168 | Not associated with survival | Bennett et al (1995) |
| IHC (DO-7) and PCR + SSCP exons 5–8 | Dukes' B = 263 Dukes' C = 278 | Not associated with survival | Soong et al (1997) |

allele often contains an inactivating mutation. Wild-type p53 is thought to be a stress response gene, the protein product of which acts to maintain genetic integrity by inducing cell cycle arrest and apoptosis following damage to the genome (Cox and Lane, 1995). According to this model, inactivation of p53 function by allelic loss, gene mutation or protein sequestration would be expected to lead to increased genetic instability and to the survival of cells with damaged DNA. Most studies of p53 protein in tumours have exploited the observation that wild-type p53 has a short half-life and is usually not detectable by immunohistochemistry, while missense gene mutations are believed to extend greatly the half-life of the protein such that it becomes detectable. Although there is a good correlation between the presence of *p53* gene mutations and positive p53 staining, discrepancies do exist and can occur for a number of reasons, including lack of protein from truncating mutations, overuse of 'antigen retrieval' methods and the criteria for classifying a tumour as 'positive' for p53 (reviewed in Wynford-Thomas, 1992; Baas et al, 1994; Hall and Lane, 1994).

A large number of studies on the association between p53 immunohistochemistry or mutational analysis and survival have been published, giving data for over 3000 patients. Immunohistochemical studies were performed using one of five different monoclonal antibodies against p53, and the criteria for 'positive' staining varied from greater than 1% to more than 10% of tumour nuclei. Several studies used two separate p53 antibodies and inconsistencies between antibodies were noted for the same tumour sections. Additional controversy also exists regarding the localization of p53. Several studies have reported the presence of cytoplasmic staining for p53, which they report to be of greater prognostic significance than the more commonly observed nuclear staining (Sun et al, 1992; Bosari et al, 1994). It is unclear whether the cytoplasmic staining represents a methodological artifact or a finding of biological and/or prognostic importance.

The wide availability of antibodies for immunohistochemistry of p53 in formalin-fixed tissue has led to its use in the majority of studies assessing the association between p53 and survival (Bosari and Viale, 1995; reviewed in Manne et al, 1997). The role of p53 staining as a prognostic tool in colorectal cancer has been clouded by a similar number of studies (and patient numbers) that have reported poor survival in p53-positive cases as demonstrating no association between p53 overexpression and outcome (Table 3). This conflict in prognostic importance exists both in studies focusing on specific stage of disease and in those including Dukes' stage A–D. Although the sample size of the majority of studies was 100–200 patients, the presence of several larger studies (250–300) has not clarified the clinical relevance of p53 immunohistochemistry.

Three out of four studies of p53 and survival in advanced colorectal cancer found no association (Brett et al, 1996; Paradiso et al, 1996; Costa et al, 1997), with the exception being an evaluation of the impact in the context of intrahepatic chemotherapy for liver metastasis (Belluco et al, 1996). A single study found no association between either p53 staining or *ras* mutations and survival, but identified a poor prognosis in patients whose tumour contained both alterations (Bell et al, 1993). Additional studies identified additive prognostic significance when p53 and bcl-2 alterations were present in the same patient (Barreton et al, 1996*a*; Manne et al, 1997). This suggests the need for several large, prospective studies (>500 patients) with long clinical follow-up (>5 years) and a uniform treatment policy to provide definitive data on the interplay between p53 and other putative prognostic markers to identify patients with a poor prognosis for whom more aggressive therapy may be appropriate.

Molecular analysis used either direct DNA or cDNA sequencing or single-strand conformational polymorphism (SSCP) and focused on *p53* exons 5–8, where 98% of mutations have been described. The concordance between immunohistochemical and molecular analysis is 60–85%, with both false-positive and false-negative staining observed.

Studies of mutations in *p53* have also been conflicting, with the majority showing no association with patient survival (Goh et al, 1995; Leahy et al, 1996; Pricolo et al, 1996; Soong et al, 1997) (Table 3). The studies showing no association had longer patient follow-up, suggesting that the effort of tumour microdissection and the time and expense of *p53* sequencing or SSCP is not a worthwhile exercise for identifying patients at risk of poor survival.

The largest study of p53 alterations and patient prognosis (541 patients) used both immunohistochemistry and PCR-SSCP and had a mean patient follow-up time of over 7 years (Soong et al, 1997). Although no association with prognosis was identified from the molecular analysis, an improved survival was found in patients with an overaccumulation of p53. This puzzling finding was strongest for distal colonic tumours and patients with Dukes' C disease. However, even in the left-sided tumours, the improvement in patient survival associated with p53 staining was 10–20%. This smaller degree of survival advantage may help to explain why many smaller studies of mixed tumour stages have found no statistically significant prognostic importance for p53. Only a small number of patients in the study received post-operative

© Cancer Research Campaign 1999

chemotherapy, restricting the application of these data to current treatment protocols.

Studies in cell lines with a homozygous mutant *p53* genotype demonstrated a high degree of resistance to radiation and several chemotherapy agents, including 5-FU (Lowe et al, 1993). This provided the basis for four studies (a total of 222 patients) of the association between p53 staining and response to either systemic or intrahepatic 5-FU-based chemotherapy for advanced colorectal cancer (Belluco et al, 1996; Brett et al, 1996; Paradiso et al, 1996) or combined chemoradiation neoadjuvant therapy in rectal cancer (Spitz et al, 1997). Immunohistochemistry was performed in formalin-fixed tissues using either monoclonal antibody 1801 (Belluco et al, 1996; Paradiso et al, 1996) or DO-1 (Brett et al, 1996; Spitz et al, 1997) and the criteria for a p53-positive tumour ranged from greater than 1% to greater than 10% of tumour nuclei. Tumour staining for p53 was not associated with response to either biochemically modulated (with methotrexate) or intrahepatic 5-FU therapy (Belluco et al, 1996; Paradiso et al, 1996), but a poorer response rate to 5-FU and folinic acid was observed in patients with p53 overexpression (Brett et al, 1996). Positive p53 staining in pretherapy biopsy samples was inversely correlated with a complete pathological response to chemoradiation therapy and directly correlated with an increased likelihood of residual tumour in the lymph nodes of surgical specimens (Spitz et al, 1997). This suggests that p53 staining may be a tool for identifying patients who will benefit most from neoadjuvant chemoradiation therapy or those for whom more aggressive therapy is required.

### Deleted in colon cancer/allelic variance at 18q

The deleted in colon cancer (*DCC*) gene, which is found on chromosome 18q21.2, is a putative tumour-suppressor gene with implications for tumour progression and prognosis in colorectal cancer. An important step in the Vogelstein and Fearon model of colon tumorigenesis is loss of parts of chromosome 18q, including the region containing *DCC* (Fearon and Vogelstein, 1990). Although there is conflicting information on the role of the DCC protein in tumour biology, there is evidence that it is of prognostic importance in colorectal cancer. The observed allelic loss of chromosome 18q has been linked to the prognosis of patients with stage II colorectal cancer (Jen et al, 1994). Analysis of normal and tumour DNA from microdissection of formalin-fixed, paraffin-embedded sections was performed using the dinucleotide microsatellite markers D18S58 and D18S61. No difference in prognosis was observed between patients with stage III disease and 18q loss ($n$ = 54) when compared with those with no allelic loss ($n$ = 16). However, among patients with stage II disease, the 5-year survival was 93% for those with no 18q loss ($n$ = 29) compared with 54% for those with allelic loss ($n$ = 36, $P$ = 0.006). The survival rate for stage II patients with 18q loss was not statistically different from that of patients with stage III disease. In multiple regression proportional hazards models, 18q was an independent prognostic marker and 18q loss gave a 2.5 to 3.0-fold higher risk of death. The prognostic importance of 18q allelic loss was verified in a study of 126 patients (stage I, 32; II, 50; III, 44), using four microsatellite markers on microdissected tumour (Ogunbiyi et al, 1998). Loss of 18q was an independent marker of poor prognosis, with a 1.65 relative risk of disease-free survival. Loss of 18q was prognostically important in both stage II and III disease. A poor prognosis in patients with loss of a polymorphic allele in *DCC* was also observed among 95 patients with colorectal cancer (Dukes' A, 10; B, 23; C, 47; D, 15), in which normal and tumour DNA was analysed by restriction fraction length polymorphism (RFLP) (Ishimaru et al, 1994). Loss of heterozygosity (LOH) at *DCC* gave a 5.5-fold higher risk of death and was an independent marker of survival. However, Dukes' stage was not an independent prognostic marker in the multivariate analysis.

Not all studies of 18q have demonstrated a prognostic influence. Analysis of normal and tumour DNA from 100 colorectal tumours (Dukes' B, 56; Dukes' C, 44) using RFLP found no relationship between LOH at *DCC* and survival (Dix et al, 1994). The median clinical follow-up was 18.9 months in the Dix et al (1994) study, compared with 35.4 months in the study by Jen et al (1994), and may be a reason for the discrepancy in results for this marker in patients with Dukes' B and C disease. The influence of 18q loss on prognosis has also been evaluated in metastatic colorectal cancer (Kochhar et al, 1997). LOH on 18q (microsatellite markers D18S34, D18S49, D18S35, D18S58, D18S61) was assessed in 141 patients undergoing potentially curative liver resection for colorectal cancer. The majority of tumours were non-informative for 18q markers but were informative for other loci, limiting the interpretation of this study. Among the informative cases for 18q, no relationship with either survival or disease-free survival was observed.

The use of DNA-based assessment of 18q or *DCC* is cumbersome and requires facilities for both high-quality microdissection and quality-controlled molecular biology, and therefore is unlikely to provide an approach that will have broad application to the planning of colorectal cancer treatment. There has been one published study of the prognostic implications of DCC protein staining (Shibata et al, 1996). Using a polyclonal antibody (antibody 721) and a microwave antigen retrieval protocol on formalin-fixed, paraffin-embedded tissue sections, DCC staining was evaluated in 132 patients with colorectal cancer (stage II, 70; stage III, 62) and a median follow-up of 92 months. Immunohistochemistry revealed a granular cytoplasmic staining pattern for DCC and was scored as the presence of any immunoreactivity or no evidence of staining. The expression of DCC was a prognostic factor for survival in both stage II and stage III disease. In patients with stage II disease, the 5-year survival in patients with DCC staining was 94.3% compared with 61.6% in DCC-negative tumours ($P$ < 0.001). In stage III disease, the 5-year survival was 59.3% with DCC staining compared with 33.2% in DCC-negative tumours. Expression of DCC was an independent prognostic factor which gave a threefold relative risk of death. Only tumour staging gave a similar level of prognostic information. The ease of immunohistochemical assessment of DCC will allow additional large prognostic studies to determine the role of DCC expression in the treatment planning for colorectal cancer.

### p27

The expanding understanding of cell cycle regulation has identified putative oncogenes and tumour-suppressor genes that may be involved in colorectal tumorigenesis. However, there have been few evaluations of the prognostic implications of alterations in cell cycle proteins. The cell cycle inhibitor p27 (also known as Kip 1) is a putative tumour-suppressor gene, but inactivating mutations have not been described in human tumours. Immunohistochemical analysis of p27 in formalin-fixed, paraffin-embedded sections detected variable expression in the cytoplasm, nucleus, or both, in 90% of colorectal tumours, when using a microwave-based method (Loda et al, 1997). Scoring of p27 staining was expressed as a ratio of positive cells to total number counted and all immunoreactive

© Cancer Research Campaign 1999

cells were considered positive. A significant relationship between p27 staining and prognosis was observed among 149 patients with colorectal adenocarcinoma (stage I, 31; II, 66; III, 51; IV, 1), when median survival was 151 months in patients with p27-positive tumours ($n$ = 134) and 69 months in patients with p27-negative tumours ($n$ = 15). Both tumour stage and grade showed a significant association with survival. The prognostic significance of p27 staining was independent of tumour stage and grade ($P$ = 0.003). The effect of p27 was observed when analysis was confined to patients with stage II disease, with absence of staining giving a 2.9 relative risk of death from colorectal cancer. These encouraging data now needs confirmation in a larger independent data set containing patients with uniform treatment before widespread use of p27 as a prognostic marker can be recommended.

### Allelic loss/genome damage

Although allelic instability is a common event in colorectal cancer, the prognostic impact of instability has not been extensively evaluated. Seventy-five patients (stage not reported) were evaluated for the impact of allelic deletion in chromosomes 5q and 17p on survival, using Southern blot analysis (Khine et al, 1995). Loss of chromosome 5q was observed in 32% of cases and 17p loss was found in 69%, with loss of both regions in 27% of patients and neither region in 25%. Poorer survival in patients with loss of chromosome 5q was apparent ($P$ = 0.014). Although patients with 17p allelic deletion had a poorer survival, this did not reach statistical significance ($P$ = 0.16). Multivariate analysis identified Dukes' stage and allelic loss of chromosome 5q as the two independent markers of survival ($P$ = 0.0001). An additional study evaluated allelic loss on chromosome 1p in 116 colorectal tumours, of which 82 (stage I, 17; II, 36; III, 29) were informative and evaluable for survival analysis (Ogunbiyi et al, 1997). Loss of 1p was detected in 22/82 tumours and was associated with a poorer disease-free survival. Loss of 1p was a weak but statistically significant independent marker of survival in multivariate analysis. Although the methodology used in these studies is too cumbersome for widespread application, the availability of microsatellite markers of regions throughout the genome may facilitate further evaluation of this approach for identifying patients at risk for poor long-term survival.

Genomic DNA fingerprinting has recently been used to assess the prognostic implications of tumour genome instability (Arribas et al, 1997). By comparing banding patterns from arbitrarily primed PCR analysis of 63 colorectal tumours and adjacent normal tissue, an index of genomic instability was derived and was an independent marker of prognosis. This technique does not identify specific target genes for development as markers or cellular targets for chemotherapy, but does provide an apparently reproducible method for detection of genome-wide instability for high-throughput prognostic studies. The small number of patients at each disease stage limits the conclusions from the initial study, but does provide the basis for further analysis of this methodology as a potential marker of patient survival.

### APOPTOSIS PATHWAYS

### *bcl-2*

The *bcl-2* proto-oncogene encodes a 25-kDa protein that is involved in the regulation of cell death by inhibiting apoptosis through mechanisms which are not yet defined. Its overexpression provides a growth advantage to tumour cells in vitro by blocking apoptosis. However, evaluation of bcl-2 protein expression in lung, thyroid and breast carcinomas identified a favourable prognosis in patients with overexpression of bcl-2 on tumour staining. The association between bcl-2 immunohistochemistry and patient survival has been evaluated in at least six studies of colorectal tumours (a total of 932 patients) (Bosari et al, 1995; Ofner et al, 1995; Sinicrope et al, 1995; Baretton et al, 1996*b*; Manne et al, 1997; Schneider et al, 1997). The larger studies ($n$ = 205 and 231) combined Dukes' stages A–D and found no association between bcl-2 overexpression and relapse or survival (Bosari et al, 1995; Schneider et al, 1997). In addition, bcl-2 staining was not associated with response to 5-FU-based chemotherapy (Schneider et al, 1997). Bcl-2-positive tumours were associated with a better prognosis in the remaining studies, although not always as an independent marker of overall survival (Ofner et al, 1995; Sinicrope et al, 1995; Baretton et al, 1996*b*; Manne et al, 1997). An interaction between bcl-2 overexpression and p53 staining was identified in several studies, with bcl-2-positive/p53-negative tumours being associated with a better survival than tumours with other staining patterns (Baretton et al, 1996*b*; Manne et al, 1997). This supports the need for studies of the interplay between bcl-2 and other putative prognostic markers to identify patients with a poor prognosis for whom alternative therapy may be appropriate.

bcl-2 does not act alone but forms heterodimers with other family members, including BAX, to regulate apoptosis in in vitro systems. However, there are no data on the association between BAX staining and prognosis in colorectal cancer. The recent development of techniques for the use of BAX antibodies in formalin-fixed, paraffin-embedded tumour tissue should allow this marker to be assessed in the near future (Apolinario et al, 1997; Koshida et al, 1997).

### CELL PROLIFERATION

Cell proliferation has been extensively studied using a variety of direct and surrogate markers. Currently one of the most popular methods of assessing cell proliferation is using antibodies that recognize nuclear proteins involved in, or altered during, the cell cycle. One of the most frequently studied targets is proliferating cell nuclear antigen (PCNA). This is a DNA polymerase δ accessory protein which can complex with cyclin D and cyclin-dependent kinases (i.e. p21). Ki-67 is another frequently used antibody, detecting an antigen expressed in all phases of the cell cycle except $G_0$. Evaluation of PCNA in 79 colorectal tumours (Dukes' A, 4; B, 48; C, 27) by immunohistochemistry was performed with follow-up for a minimum of 10 years (Neoptolemos et al, 1995). A PCNA score was derived by a relatively complex, subjective four-point scale according to the percentage of positive tumour cells. The PCNA index was not associated with patient survival. Sun et al (1996) studied PCNA in a series of 293 colorectal carcinomas (Dukes' A, 43; B, 101; C, 94; D, 50; stage not available for 5) which had been collected over a 15-year period and followed up for a minimum of 5 years. PCNA was assessed by counting 500 tumour nuclei and the final PCNA score was divided into four groups according to the percentage of positive nuclei. PCNA did not correlate with survival. This group also studied PCNA expression in 56 primary tumours and their corresponding lymph node metastases with good correlation between the tissues. PCNA expression was investigated in 71 advanced colorectal cancers (metastatic, $n$ = 33, or recurrent disease, $n$ = 38) eligible to receive

 © Cancer Research Campaign 1999

5-FU-based therapy (Paradiso et al, 1996). PCNA was scored by analysing 1000 nuclei, and a 'high' PCNA score (greater than 32% positive cells) was associated with improved survival and was independently prognostic.

### ANGIOGENESIS/METASTASIS/INVASION

### Thymidine phosphorylase

Thymidine phosphorylase, or platelet-derived endothelial cell growth factor, is an enzyme that catalyses the reversible phosphorylysis of thymidine and deoxyuridine to their bases and deoxyribose phosphate. It has been shown to be capable of inducing angiogenesis, and expression of thymidine phosphorylase may also be important for the activation of certain prodrugs, for example deoxyfluorouridine conversion to fluorouracil (Griffiths and Stratford, 1997). Thymidine phosphorylase was evaluated in 93 colorectal cancers (Dukes' stage A, 30; B, 42; C, 21) by immunohistochemistry using a monoclonal antibody (Takebayashi et al, 1996). The immunohistochemical assessment used was a straightforward two-point scale, with tumour being classified as positive if more than 5% of tumour cells displayed staining. Normal colon mucosa did not stain, whereas staining was observed in both tumour and stromal cells. No comment was made regarding the significance of stromal cell staining in the absence of tumour cell staining or how this was scored. The presence of thymidine phosphorylase was associated with a significantly poorer prognosis. Although expression correlated with other histopathological criteria (lymph node metastasis, lymphatic and venous invasion), thymidine phosphorylase also appeared to be an independent prognostic factor.

### Vascular endothelial growth factor

Vascular endothelial growth factor (VEGF) is a glycoprotein that shares some homology with platelet-derived growth factor and is intimately involved in promoting angiogenesis. Interest in angiogenesis has grown because of the possibility of using anti-angiogenic therapy to prevent tumour progression. There have been several recent reports of the analysis of VEGF in colorectal cancer, all of which used immunohistochemistry on formalin-fixed, wax-embedded sections and showed that VEGF is primarily localized to tumour cells. In the largest study of 175 colorectal carcinomas (stage 0, 7; I, 24; II, 48; III, 59; IV, 37), the presence of VEGF correlated with increasing tumour stage and was associated with decreased overall survival ($P < 0.05$) and decreased disease-free survival ($P < 0.01$) (Kang et al, 1997). However, it was not an independent prognostic factor. Amaya et al (1997) studied the presence of VEGF in 133 cases of colorectal cancer (stage I, 14; II, 42; III, 45; IV, 29 with three unclassified cases). VEGF was localized to tumour cells, and VEGF-positive tumours had a significantly poorer prognosis than VEGF-negative tumours ($P < 0.02$), which remained significant in a multivariate analysis. A much smaller study of VEGF in 27 Dukes' stage B patients showed essentially similar results (Takahashi et al, 1997).

### Matrix metalloproteinases and inhibitors

The matrix metalloproteinases (MMPs) are a group of enzymes with a central role in matrix degradation, and there is considerable evidence to indicate that this group of enzymes have a key role in tumour invasion and metastasis. The current concept of the MMPs in tumour invasion is that they not only have a direct role in facilitating extracellular matrix degradation but as a consequence they also have an important role maintaining the tumour environment and thus promoting tumour growth (Chambers and Matrisian, 1997). There are currently at least 15 known MMPs, which are broadly classified into collagenases, gelatinases, stromelysins and membrane-type MMPs. The MMPs (except membrane-type MMPs) are produced as proenzymes and are activated by cleavage of N-terminal propeptides. Our group identified MMP-1 (interstitial collagenase) in 64 patients with colorectal cancers (Dukes' A, 1; B, 38; C, 25). The presence of MMP-1 in tumour cells was associated with poorer prognosis and was independent of Dukes' stage (Murray et al, 1996). This study used a simple assessment of the presence or absence of MMP-1 immunoreactivity and is likely to be more reproducible than some of the more complex scoring schemes used in other immunohistochemistry studies. MMP-9 mRNA has been identified by in situ hybridization and Northern blotting in 71 tumours (Dukes' A, 11; B, 18; C, 16; D, 26), and a high ratio of tumour to normal MMP-9 mRNA was associated with poor prognosis and was independent of Dukes' stage (Zeng et al, 1996). In a small group ($n = 31$) of colorectal cancers, total type IV collagenase activity (MMP-2 and MMP-9) has been found to be prognostically significant, with high activity associated with both shorter disease-free survival and shorter overall survival (Ambiru et al, 1997). These findings of the different MMPs emphasize the need to perform a comprehensive series of studies of MMPs in colorectal cancer to understand the interactions between the individual MMPs and to identify which of the MMPs are most appropriate as prognostic markers. Studies are also required to determine whether it is more relevant to assess total enzyme or active enzyme. Clinical trials of MMP inhibitors are now under way and require evaluation in relation to tumour MMP expression to determine if MMP expression is a reliable method of selecting patients for anti-MMP therapy. The MMPs are inhibited in vivo by naturally occurring inhibitors, tissue inhibitors of metalloproteinases (TIMPs). There are currently four known TIMPs, and studies are also necessary to determine the contribution of TIMPs to prognosis.

### Urokinase-type plasminogen activator and inhibitor

Urokinase-type plasminogen activator (uPA) is a serine protease which catalyses the activation of plasminogen to plasmin, a broad-spectrum protease which in turn can break down a variety of extracellular matrix components and also promote the activation of MMP-2. In a series of Dukes' B colon cancers ($n = 70$), a higher proportion of uPA-positive tumour cells by immunohistochemistry was associated with poorer survival ($P = 0.01$) (Mucalhy et al, 1994). However, most tumours had a high percentage of positive tumour cells (57/70) and only 13 cases had a low percentage of positive tumour cells. In addition, stromal staining for uPA showed an association with survival. This study is one of relatively few to study a marker in relation to a single Dukes' stage. The patients in this study all had uniform treatment with no post-operative radio- or chemotherapy and follow-up was for between 5 and 10 years. High tumour levels of plasminogen activator inhibitor-2 (PAI-2) in Dukes' B ($n = 82$) and C ($n = 54$) colorectal cancers has also been found to be prognostically significant ($P = 0.05$), but not independent of Dukes' stage, in a group of 136 colorectal cancers (Ganesh et al, 1997).

### CD44

CD44 is a cell-surface glycoprotein which functions as an adhesion molecule. Multiple isoforms of CD44 are generated by alternative splicing of up to ten exons that encode for the molecule's extracellular portion. CD44 variants have been identified on the surface of a variety of tumour cells. CD44v6 was studied by immunohistochemistry in a series of colon cancers ($n$ = 68) with follow-up of between 6.5 and 9.5 years (Mulder et al, 1994). The report did not indicate the distribution of tumours in different Dukes' stages or the post-operative treatments received by the patients. Tumours that strongly expressed CD44v6 had a poorer prognosis than those tumours that showed less intense or no immunostaining. This was stated to be independent of Dukes' stage. CD44 exons 8–10 was studied using immunohistochemistry in 215 colorectal tumours (stage I, 37; II, 71; III, 85; IV, 22) (Yamaguchi et al, 1996). There was no significant immunoreactivity for CD44 in normal mucosa while there was positive immunoreactivity in tumour cells. Immunoreactivity was assessed as either present or absent, although the cut-off point for negative score was less than 25% of tumour cells. The presence of CD44 was associated with poorer prognosis, and in multivariate analysis this factor remained independently prognostic.

### E-cadherin

E-cadherin is a member of the cadherin family of calcium-dependent adhesion molecules that function as mediators of cell-to-cell adhesion. It is a transmembrane protein with a large extracellular domain and a smaller cytoplasmic component which interacts with cytoplasmic proteins, particularly β-catenin. Loss of E-cadherin is associated with increased cell invasiveness. E-cadherin was studied by in situ hybridization in 49 Dukes' B colorectal cancers (Dorudi et al, 1995). In situ hybridization was performed because antibodies which reliably detect E-cadherin in formalin-fixed, wax-embedded sections do not appear to be currently available. The lowest expression of E-cadherin was associated with poor survival. In a second study, E-cadherin expression was investigated by in situ hybridization in 32 colorectal cancers (Dukes' A, B and D). None of the patients had received chemotherapy and all had been followed up for at least 5 years (Kitadai et al, 1996). The profile of expression of E-cadherin was also compared with several other genes involved in tumour invasion, including type IV collagenase. There was no prognostic significance of E-cadherin expression, although low levels of E-cadherin and a high ratio of type IV collagenase to E-cadherin were associated with an increased incidence of metastasis.

### *Helix pomatia* agglutinin

*Helix pomatia* agglutinin (HPA) is a sugar-binding protein, or lectin, which binds specifically to *N*-acetylgalactosamine residues. The binding of this lectin has been suggested to be of prognostic significance in breast cancer. HPA binding was studied in 130 colorectal cancers (Dukes' stage A, 14; B, 49; C, 62; unknown, 5). Using a histochemical approach to detect binding of this lectin (Schumacher et al, 1994). HPA binding correlated with Dukes' stage, but was not independently prognostic.

### INTRACELLULAR TARGETS OF CHEMOTHERAPY

### Thymidylate synthase

Thymidylate synthase (TS) is the primary intracellular target for two of the more commonly used chemotherapeutic agents for colorectal cancer, 5-FU and raltitrexed (tomudex). Elevated expression of the TS protein has been demonstrated in cell lines which were made resistant to either 5-FU or tomudex (Johnston et al, 1992; Jackman et al, 1995). A higher degree of tumour TS mRNA and protein was observed in patients with a poor response to 5-FU in a small study of colorectal ($n$ = 9) and gastric ($n$ = 12) cancers (Johnston et al, 1995). TS mRNA expression provided a clearer definition of patient response than did protein level in this study. Quantitative PCR assay of TS mRNA was then applied to tumour tissue from 46 patients with advanced colorectal cancer receiving protracted infusion of 5-FU (200 mg $m^{-2}$ per day for 21 days) and weekly leucovorin (Leichman et al, 1997). No patient with a ratio of TS to β-actin (used as an intertumour control) greater than $4.1 \times 10^{-3}$ responded. Using the median TS/β-actin ratio as a cut-off point, survival was significantly higher (median 13.6 months) in patients with less than median TS levels than in those with greater than median values (8.2 months) (Leichman et al, 1997). Similar results have recently been reported in patients receiving hepatic artery infusion of fluoropyrimidines, when patients with low TS mRNA expression were 4.1-fold more likely to respond to chemotherapy (Kornmann et al, 1997). TS antibodies, which work on formalin-fixed, paraffin-embedded tumour tissue, have provided the opportunity for retrospective analysis of the prognostic significance of TS staining in patients with colorectal cancer. TS was assessed with a semiquantitative visual grading system based on the intensity of staining (0 to +++) and extent of staining (focal, <25% positive cells; diffuse, >25% positive cells) (Johnston et al, 1994). An initial study in 294 patients (Dukes' stage A, 32; B, 92; C, 146; D, 24) from NSABP study R-01 demonstrated that high expression of TS protein in tumour is associated with a poor survival rate in patients receiving 5-FU-based adjuvant chemotherapy for rectal cancer (Johnston et al, 1994). TS staining intensity was an independent prognostic marker and identified a significant impact of 5-FU-based chemotherapy in patients with high TS levels. However, TS staining did not predict disease response among 134 patients receiving 5-FU-based chemotherapy for advanced colorectal cancer (Findlay et al, 1997). A significant time interval between surgical resection of the primary tumour and treatment of advanced disease was observed for most patients. As this study performed TS staining on the primary tumour only, this may point to clinically significant differences between TS staining in primary and metastatic tumour. Although TS staining has demonstrated potential prognostic significance, prospective studies are required to confirm the importance in adjuvant chemotherapy and to determine the role of TS levels in advanced colorectal cancer.

### GENERAL MARKERS

### Carcinoembryonic antigen

Carcinoembryonic antigen is often used as a serum marker in follow-up studies of colorectal cancer to detect recurrences/metastasis. However, generally, the detection of CEA staining in colorectal tumour samples has not been found to be useful in providing prognostic information.

### Sialyl Lewis$^x$ antigen

The sialyl Lewis antigen ($sLe^x$) is a cell-surface glycoprotein which is part of the blood group system of antigens and may serve as a ligand for endothelial leucocyte adhesion molecule. This may

*© Cancer Research Campaign 1999*

facilitate the binding of tumour cells to endothelial cells, which is a step in the metastatic cascade. High expression of this antigen is associated with increased tumour cell invasion. The presence of $sLe^x$ in 132 colorectal cancers (stage I, 36; II, 40; III, 38; IV, 18) was evaluated by immunohistochemistry on formalin-fixed, wax-embedded sections with a monoclonal antibody to this antigen (Nakamori et al, 1993). Tumours with greater than 5% of positive cells were assessed as positive and those with less than 5% positive cells were scored as negative. Follow-up was between 5 and 7 years. The presence of the $sLe^x$ was associated with depth of tumour invasion, lymphatic invasion, lymph node metastasis and tumour stage. Overall and disease-free 5-year survival was significantly less in the $sLe^x$-positive tumours, and this factor was also independently prognostic for both overall survival and disease-free survival.

### CONCLUSIONS

Although the difficulty in identifying markers of prognosis has led to much scepticism, there is still a real need for methodologies to guide the choice of therapy and use health care expenditure in a more rational manner for this common disease. Future studies must focus on multiple markers in large numbers of patients at a single disease stage in order to provide definitive evidence on the clinical utility of prognostic markers and their relative contribution to the prediction of survival. The recent emphasis by the National Cancer Institute on pathology archives linked to large therapeutic studies as a resource for collaborative research is an example of the way funding can be used to answer these important questions about patient care.

### ACKNOWLEDGEMENTS

Research in the authors' laboratories are supported in part by an Aberdeen Colorectal Cancer Initiative grant from the University of Aberdeen Development Trust. The helpful comments of Professor J Cassidy, and Dr J McKay were greatly appreciated. Many thanks to Maria Johnson for her secretarial skills.

### REFERENCES

Amaya H, Tanigawa N, Lu C, Matsumura M, Shimomatsuya T, Horiuchi T and Muroaka R (1997) Association of vascular endothelial growth factor expression with tumor angiogenesis, survival and thymidine phosphorylase/platelet-derived endothelial cell growth factor expression in human colorectal cancer. *Cancer Lett* **119**: 227–235

Ambiru S, Miyazaki M, Ito H, Nakagawa K, Shimizu H, Nukui Y, Nozawa S, Okuno A, Yoshitomi H and Nakajima N (1997) A prospective study of prognostic value of type IV collagenase in colorectal cancer tissue. *Dig Dis Sci* **42**: 1660–1665

Anderson SN, Lovig T, Breivik J, Lund E, Guadernack G, Meling GI and Rognum TO (1996) K-ras mutations and prognosis in large-bowel carcinomas. *Scand J Gastroenterol* **32**: 62–69

Apolinario RM, van der Valk P, de Jong JS, Deveille W, Van Arkotte J, Dingemans A-MC, Van Mourik JC, Postmus PE, Pinedo HM and Giaccone G (1997) Prognostic value of the expression of p53, *bcl*-2, and *bax* oncoproteins, and neovascularization in patients with radically resected non-small-cell lung cancer. *J Clin Oncol* **15**: 2456–2466

Arribas R, Capellà G, Tórtola S, Masramon L, Grizzle WE, Perucho M and Peinado MA (1997). Assessment of genomic damage in colorectal cancer by DNA fingerprinting: prognostic applications. *J Clin Oncol* **15**: 3230–3240

Astler VB and Coller FA (1954) The prognostic significance of direct extension of carcinoma of the colon and rectum. *Ann Surg* **192**: 846–850

Augenlicht LH, Wadler S, Corner G, Richards C, Ryan L, Multani AS, Pathak S, Benson A, Haller D and Heerdt BG (1997) Low-level *c-myc* amplifications in human colonic carcinoma cell lines and tumours: a frequent, p53-independent mutation associated with improved outcome in a randomized multi-institutional trial. *Cancer Res* **57**: 1769–1775

Baas IO, Mulder JR, Offerhaus GJA, Vogelstein B and Hamilton SR (1994) An evaluation of six antibodies for immunohistochemistry of mutant p53 gene product in archival colorectal neoplasms. *J Pathol* **172**: 5–12

Baretton GB, Vogt M, Muller C, Diebold J, Schneiderbanger K, Schmidt M and Lohrs U (1996*a*) Prognostic significance of p53 expression, chromosome 17 copy number, and DNA ploidy in non-metastasized colorectal carcinomas (stages IB and II). *Scand J Gastroenterol* **31**: 481–489

Baretton GB, Diebold J, Christoforis G, Vogt M, Müller C, Dopfer K, Schneiderbanger K, Schmidt M and Löhrs U (1996*b*) Apoptosis and immunohistochemical bcl-2 expression in colorectal adenomas and carcinomas. Aspects of carcinogenesis and prognostic significance. *Cancer* **77**: 255–264

Bartik Z, Nordenskiöld BO and Sun Z-F (1997) p53 overexpression as a prognostic factor in patients with Dukes' B colorectal adenocarcinoma. *Int J Oncol* **11**: 1019–1023

Bell SM, Scott N, Cross D, Sagar P, Lewis FA, Blair GE, Taylor GR, Dixon MF and Quirke P (1993) Prognostic value of p53 overexpression and c-Ki-ras gene mutations in colorectal cancer. *Gastroenterology* **104**: 57–64

Belluco C, Guillem JG, Kemeny N, Huang Y, Klimstra D, Berger MF and Cohen AM (1996) p53 Nuclear protein overexpression in colorectal cancer: a dominant predictor of survival in patients with advanced hepatic metastases. *J Clin Oncol* **14**: 2696–2701

Benhattar J, Losi L, Chaubert P, Givel J-C and Costa J (1993) Prognostic significance of K-ras mutations in colorectal carcinoma. *Gastroenterology* **104**: 1044–1048

Bennett MA, Kay EW, Mulcahy H, O'Flaherty L, O'Donoghue, DP, Leader M and Croke DT (1995) ras and p53 in the prediction of survival in Dukes' stage B colorectal carcinoma. *J Clin Pathol* **48**: M310–M315

Bhatavdekar JM, Patel DD, Ghosh N, Chikhlikar PR, Trivedi TI, Suthar TP, Doctor SS, Shah NG and Balar DB (1997) Coexpression of Bcl-2, c-Myc, and p53 oncoproteins as prognostic discriminants in patients with colorectal carcinoma. *Dis Colon Rectum* **40**: 785–790

Bosari S and Viale G (1995) The clinical significance of p53 aberrations in human tumors. *Virchows Arch A Pathol Anat Histopathol* **427**: 229–241

Bosari S, Viale G, Bossi P, Maggioni M, Coggi G, Murray JJ and Lee AKC (1994) Cytoplasmic accumulation of p53 protein: an independent prognostic indicator in colorectal adenocarcinomas. *J Natl Cancer Inst* **86**: 681–687

Bosari S, Moneghini L, Graziani D, Lee AKC, Murray JJ, Coggi G and Viale G (1995) Bcl-2 oncoprotein in colorectal hyperplastic polyps, adenomas, and adenocarcinomas. *Human Pathol* **26**: 534–540

Brett MC, Pickard M, Green B, Howel-Evans A, Smith D, Kinsella A and Poston G (1996) p53 protein overexpression and response to biomodulated 5-fluorouracil chemotherapy in patients with advanced colorectal cancer. *Eur J Sur Oncol* **22**: 182–185

Chambers AF and Matrisian LM (1997) Changing views of the role of matrix metalloproteinases in metastasis. *J Natl Cancer Inst* **89**: 1260–1270

Cohen AM, Tremiterra S, Candela F, Thaler HT and Sigurdson ER (1991) Prognosis of node-positive colon cancer. *Cancer* **67**: 1859–1861

Costa A, Doci R, Machen C, Bignami P, Faranda A, Gennari L and Silvestrini R (1997) Cell proliferation-related markers in colorectal liver metastases: correlation with patients prognosis. *J Clin Oncol* **15**: 2008–2014

Cox LS and Lane DP (1995) Tumor suppressors, kinases and clamps – how p53 regulates the cell-cycle in response to DNA-damage. *Bioessays* **17**: 510–508

Dix BR, Robbins P, Soong R, Jenner D, House AK, Lacopetta BJ, and The General Surgeons at Sir Charles Gairdner Hospital (1994) The common molecular genetic alterations in Dukes' B and C colorectal carcinomas are not short-term prognostic indicators of survival. *Int J Cancer* **59**: 747–751

Dorudi S, Hanby AM, Poulsom R, Northover J and Hart IR (1995) Level of expression of E-cadherin mRNA in colorectal cancer correlates with clinical outcome. *Br J Cancer* **71**: 614–616

Elnatan J, Goh H-S and Smith DR (1996) C-KI-RAS activation and the biological behaviour of proximal and distal colonic adenocarcinomas. *Eur J Cancer* **32A**: 491–497

Fearon ER and Vogelstein BA (1990) The genetic model for colorectal tumorigenesis. *Cell* **61**: 759–767

Findlay MPN, Cunningham D, Morgan G, Clinton S, Hardcastle A and Aherne GW (1997) Lack of correlation between thymidylate synthase levels in primary colorectal tumours and subsequent response to chemotherapy. *Br J Cancer* **75**: 903–909

Ganesh S, Sier CFM, Heerding MM, Vankrieken JHJM, Griffioen G, Welvaart K, van de Velde CJ, Verheijen JH, Lamers, CB and Verspaget HW (1996) Contribution of plasminogen activators and their inhibitors to the survival

© Cancer Research Campaign 1999 

prognosis of patients with Dukes' stage B and C colorectal cancer. *Br J Cancer* **75**: 1793–1801

Goh H-S, Yao J and Smith DR (1995) p53 Point mutation and survival in colorectal cancer patients. *Cancer Res* **55**: 5217–5221

Griffiths L and Stratford IJ (1997) Platelet-derived endothelial cell growth factor thymidine phosphorylase in tumour growth and response to therapy. *Br J Cancer* **76**: 689–693

Hall PA and Lane DP (1994) p53 in tumour pathology: can we trust immunohistochemistry? – Revisited! *J Pathol* **172**: 1–4

Hermanek P, Wiebelt H, Staimner D and Riedl S (1995) Prognostic factors of rectum carcinoma – experience of the German multicentre study SGCRC. German Study Group Colo-Rectal Carcinoma. *Tumori* **81**: 60–64

Hoffmann D, Moore J and Roder D (1997) Trends in survival from colonic cancer: The impact of subspecialization. *Aus N Z J Surgery* **67**: 842–845

Ishimuru G, Ookawa K, Yamaguchi N, Sakamoto M, Hirohashi S, Muto T and Yokota J (1994) Allelic losses associated with the metastatic potential of colorectal carcinoma. *Int J Oncol* **5**: 267–273

Jackman AL, Kelland LR, Kimbell R, Brown M, Gibson W, Aherne GW, Hardcastle A and Boyle FT (1995) Mechanisms of acquired resistance to the quinazoline thymidylate synthase inhibitor ZD1694 (tomudex) in one mouse and three human cell lines. *Br J Cancer* **71**: 914–924

Jen J, Kim HG, Piantadosi S, Liu ZF, Lecitt RC, Sistonen P, Kinzler KW, Vogelstein B and Hamilton SR (1994) Allelic loss of chromosome 18q and prognosis in colorectal cancer. *N Engl J Med* **331**: 213–221

Johnston PG, Drake JC, Trepel J and Allegra CJ (1992) Immunological quantitation of thymidylate synthase using the monoclonal antibody TS 106 in 5-fluorouracil-sensitive and -resistant human cancer cell lines. *Cancer Res* **52**: 4306–4312

Johnston PG, Fisher ER, Rockette HE, Fisher B, Wolmark N, Drake JC, Chabner BA and Allegra CJ (1994) The role of thymidylate synthase expression in prognosis and outcome of adjuvant chemotherapy in patients with rectal cancer. *J Clin Oncol* **12**: 2640–2647

Johnston PG, Lenz H-J, Leichman CG, Danenberg KD, Allegra CJ, Danenberg PV and Leichman L (1995) Thymidylate synthase gene and protein expression correlate and are associated with response to 5-fluorouracil in human colorectal and gastric tumours. *Cancer Res* **55**: 1407–1412

Kang SM, Maeda K, Chung YS, Onodda N, Ogawa Y, Takatsuka S, Ogawa M, Sawada T, Nakata B, Nishiguchi Y, Ikehara T, Okuno M and Sowa M (1997) Vascular endothelial growth factor expression correlates with hematogenous metastasis and prognosis in colorectal carcinoma. *Oncol Rep* **4**: 381–384

Kapitanovic S, Radosevic S, Kapitanovic M, Ajndelinovic S, Ferencic Z, Tavassoli M, Primorac D, Sonicki Z, Spaventi S, Pavelic K and Spaventi R (1997) The expression of p185$^{HER-2/neu}$ correlates with the stage of disease and survival in colorectal cancer. *Gastroenterology* **112**: 1103–1113

Kay EW, Mulcahy H, Walsh CB, Leader M and O'Donoghue D (1994) Cytoplasmic *c-erb* B2 protein expression correlates with survival in Dukes' B colorectal carcinoma. *Histopathology* **25**: 455–461

Khine K, Goh H-S and Smith DR (1995) Prognostic significance of chromosome 5q and 17p allelic deletion in colorectal adenocarcinomas. *Int J Oncol* **7**: 631–635

Kitadai Y, Ellis LM, Tucker SL, Greene GF, Bucana CD, Cleary KR, Takahashi Y, Tahara E and Fidler IJ (1996) Multiparametric in situ mRNA hybridization analysis to predict disease recurrence in patients with colon carcinoma. *Am J Path* **149**: 1541–1551

Kochhar R, Halling KC, McDonnell S, Schaid DJ, French AJ, O'Connell MJ, Nagorney DM and Thibodeau SN (1997) Allelic imbalance and microsatellite instability in resected Dukes' D colorectal cancer. *Diagn Mol Pathol* **6**: 78–84

Kornmann M, Link KH, Lenz H-J, Pillasch J, Metzger R, Butzer U, Leder GH, Weindel M, Safi F, Danenberg KD, Beger HG and Danenberg PV (1997) Thymidylate synthase is a predictor for response and resistance in hepatic artery infusion chemotherapy. *Cancer Lett* **118**: 29–35

Koshida Y, Saegusa M and Okayasu I (1997) Apoptosis, cell proliferation and expression of Bcl-2 and Bax in gastric carcinomas: immunohistochemical and clinicopathological study. *Br J Cancer* **75**: 367–373

Kressner ULF, Lindmark G, Gerdin B, Pahlman L and Glimelius B (1996) Immunohistochemical p53 staining is of limited value in the staging and prognostic prediction of colorectal cancer. *Anticancer Res* **16**: 951–958

Kumar V, Bustin SA and Mckay IA (1995) Transforming growth factor-alpha. *Cell Biol Int* **19**: 373–388

Lanza G, Maestri I, Dubini A, Gafa R, Santini A, Ferretti S and Cavazzini L (1996) P53 expression in colorectal cancer. Relation to tumor type, DNA ploidy pattern, and short-term survival. *Am J Clin Pathol* **105**: 604–612

Lawrence DA (1996) Transforming growth factor-beta – a general review. *Eur Cytokine Network* **7**: 363–374

Leahy DT, Salman R, Mulcahy H, Sheahan K, O'Donoghue DP and Parfrey NA (1996) Prognostic significance of p53 abnormalities in colorectal carcinoma detected by PCR-SSCP and immunohistochemical analysis. *J Pathol* **180**: 364–370

Leichman CG, Lenz H-J, Leichman L, Danenberg K, Baranda J, Groshen S, Boswell W, Metzger R, Tan M and Danenberg PV (1997) Quantitation of intratumoral thymidylate synthase expression predicts for disseminated colorectal cancer response and resistance to protracted-infusion fluorouracil and weekly leucovorin. *J Clin Oncol* **15**: 3223–3229

Linden MD, Nathanson DS and Jacobson G (1996) Lack of prognostic significance of epidermal growth factor receptor expression in Dukes' B colorectal carcinomas. *Lab Invest* **74**: 61A:343

Loda M, Cukor B, Tam SW, Lavin P, Fiorentino M, Draetta GF, Jessup JM and Pagano M (1997) Increased proteasome-dependent degradation of the cyclin dependent kinase inhibitor p27 in aggressive colorectal carcinomas. *Nature Med* **3**: 231–234

Lowe SW, Ruley HE, Jacks T and Housman DE (1993) p53-dependent apoptosis modulates the cytotoxicity of anticancer agents. *Cell* **74**: 957–967

Manne U, Myers RB, Moron C, Poczatek RB, Dillard S, Weiss H, Brown D, Srivastava S and Grizzle WE (1997) Prognostic significance of Bcl-2 expression and p53 nuclear accumulation in colorectal adenocarcinoma. *Int J Cancer* **74**: 346–358

Markowitz S, Hines JD, Lutterbaugh J, Myeroff L, Mackay W, Gordon N, Rustum Y, Luna E and Kleinerman J (1995) Mutant K-ras oncogenes in colon cancers do not predict patient's chemotherapy response or survival. *Clin Cancer Res* **1**: 441–445

Mayer A, Takimoto M, Fritz E, Schellander G, Kofler K and Ludwig H (1993) The prognostic significance of proliferating cell nuclear antigen, epidermal growth factor receptor, and mdr gene expression in colorectal cancer. *Cancer* **71**: 2454–2460

McArdle CS and Hole D (1991) Impact of variability among surgeons on post-operative morbidity and mortality and ultimate survival. *Br Med J* **302**: 1501–1505

Miller F, Heimann TM, Quish A, Pyo DJ, Szporn A, Martinelli G and Fasy TM (1992) Ras and c-myc protein expression in colorectal carcinoma. *Dis Colon Rectum* **35**: 430–435

Miyahara M, Saito T, Kaketani K, Sato K, Kuwahara A, Shimoda K and Kobayashi M (1991) Clinical significance of ras p21 overexpression for patients with an advanced colorectal cancer. *Dis Colon Rectum* **34**: 1097–1102

Moertel CG, Fleming TR, MacDonald JS, Haller DG, Laurie JA, Tangen CM, Ungerleider JS, Emerson WA, Tormey DC, Glick JH, Veeder MH, Mailliard JA and Graff J (1995) Fluorouracil plus levamisole as effective adjuvant therapy after resection of stage III colon carcinoma: a final report. *Ann Intern Med* **122**: 321–326

Mulcahy HE, Duffy MJ, Gibbons D, McCarthy P, Parfrey NA, O'Donoghue DP and Sheahan K (1994) Urokinase type plasminogen activator and outcome in Dukes' B colorectal cancer. *Lancet* **344**: 583–584

Mulder JWR, Kruyt PM, Sewnath M, Oosting J, Seldenrijk CA, Weidema WF, Offerhaus GJ and Pals ST (1994) Colorectal cancer prognosis and expression of exon-v6 containing CD44 proteins. *Lancet* **344**: 1470–1472

Murray GI, Duncan ME, O'Neil P, Melvin WT and Fothergill JE (1996) Matrix metalloproteinase-1 is associated with poor prognosis in colorectal cancer. *Nature Med* **2**: 461–462

Nagai MA, Habr-Gama A, Oshima BS and Bretani MM (1992) Association of genetic alterations of c-myc, c-fos, and c-Ha-ras proto-oncogenes in colorectal tumors. Frequency and clinical significance. *Dis Colon Rectum* **35**: 444–451

Nakamori S, Kameyama M, Imaoka S, Furukawa H, Ishikawa O, Sasaki Y, Kabuto T, Iwanaga T, Matsushita Y and Irimura T (1993) Increased expression of Sialyl Lewis antigen correlates with poor survival in patients with colorectal carcinoma: clinicopathological and immunohistochemical study. *Cancer Res* **53**: 3632–3637

Neoptolemos JP, Oates GD, Newbold KM, Robson AM, McConkey C and Powell J (1995) Cyclin/proliferation cell nuclear antigen immunohistochemistry does not improve the prognostic power of Dukes' or Jass' classifications for colorectal cancer. *Br J Surg* **82**: 184–187

Öfner D, Riehemann K, Maier H, Riedmann B, Nehoda H, Tötsch M, Böcker W, Jasani B and Schmid KW (1995) Immunohistochemically detectable bcl-2 expression in colorectal carcinoma: correlation with tumour stage and patient survival. *Br J Cancer* **72**: 981–985

Ogunbiyi OA, Goodfellow PJ, Gagliardi G, Swanson PE, Birnbaum EH, Fleshman JW, Kodner IJ and Moley JF (1997) Prognostic value of chromosome 1 p allelic loss in colon cancer. *Gastroenterology* **113**: 761–766

Ogunbiyi OA, Goodfellow PJ, Herfarth K, Gagliardi G, Swanson PE, Birnbaum EH, Read TE, Fleshman JW, Kodner IJ and Moley JF (1998) Confirmation that

 *© Cancer Research Campaign 1999*

chromosome 18q allelic loss in colon cancer is a prognostic indicator. *J Clin Oncol* **16**: 427–433
Paradiso A, Rabinovich M, Vallejo C, Machiavelli M, Romero A, Perez J, Lacava J, Cuevas MA, Rodriquez R, Leone B, Sapia MG, Simone G and De Lena M (1996) p53 and PCNA expression in advanced colorectal cancer: response to chemotherapy and long-term prognosis. *Int J Cancer* **69**: 437–441
Parkin DM, Pisani P and Ferlay J (1993) Estimates of the worldwide incidence of eighteen major cancers in 1985. *Int J Cancer* **54**: 594–606
Poller DN, Baxter KJ and Shepherd NA (1997) p53 and Rb1 protein expression: are they prognostically useful in colorectal cancer? *Br J Cancer* **75**: 87–93
Pricolo VE, Finkelstein SD, WU T-T, Keller G, Bakker A, Swalsky PA and Bland KI (1996) Prognostic value of TP53 and K-ras-2 mutational analysis in stage III carcinoma of the colon. *Am J Surg* **171**: 41–46
Reinbach DH, McGregor JR, Murray GD and Odwyer PJ (1994) Effect of the surgeons speciality interest on the type of resection performed for colorectal-cancer. *Dis Colon Rectum* **37**: 1020–1023
Robson H, Anderson E, James RD and Schofield PF (1996) Transforming growth factor beta 1 expression in human colorectal tumours: an independent prognostic marker in a subgroup of poor prognosis patients. *Br J Cancer* **74**: 753–758
Schneider HJ, Sampson SA, Cunningham D, Norman AR, Andreyev HJN, Tilsed JVT and Clarke PA (1997) Bcl-2 expression and response to chemotherapy in colorectal adenocarcinomas. *Br J Cancer* **75**: 427–431
Schumacher U, Higgs D, Loizidou M, Pickering R, Leathem A and Taylor I (1994) Helix pomatia agglutinin binding is a useful prognostic indicator in colorectal carcinoma. *Cancer* **74**: 3104–3107
Shibata D, Reale MA, Lavin P, Silverman M, Fearon ER, Steele G Jr, Jessup JM, Loda M and Summerhayes IC (1996) The DCC protein and prognosis in colorectal cancer. *N Engl J Med* **335**: 1727–1732
Sinicrope FA, Hart J, Michelassi F and Lee JJ (1995) Prognostic value of bcl-2 oncoprotein expression in stage II colon carcinoma. *Clin Cancer Res* **1**: 1103–1110
Smith DR and Goh H-S (1996) Overexpression of the c-myc proto-oncogene in colorectal carcinoma is associated with a reduced mortality that is abrogated by point mutation of the p53 tumor suppressor gene. *Clin Cancer Res* **2**: 1049–1053
Smith DR, Elnatan J, Yao J and Goh H-S (1996) Point mutation of the c-Ki-*ras* proto-oncogene and the p53 tumour suppressor gene in distal colonic adenocarcinomas. *Int J Oncol* **8**: 1165–1169
Soong R, Grieu F, Robbins P, Dix B, Chen D, Parsons R, House A and Lacopetta B (1997) P53 alterations are associated with improved prognosis in distal colonic carcinomas. *Clin Cancer Res* **3**: 1405–1411
Span M, Moerkerk PT, De Goeij AF and Arends JW (1996) A detailed analysis of K-ras point mutations in relation to tumor progression and survival in colorectal cancer patients. *Int J Cancer* **69**: 241–245
Spitz FR, Giacco GG, Hess K, Larry L, Rich TA, Janjan N, Cleary KR and Skibber JM (1997) P53 immunohistochemical staining predicts residual disease after chemoradiation in patients with high-risk rectal cancer. *Clin Cancer Res* **3**: 1658–1690
Sun XF, Wingren S, Carstensen JM, Stål O, Hatschek T, Boeryd B, Nordenskjöld B and Zhang H (1991) Ras p21 expression in relation to DNA ploidy, s-phase fraction and prognosis in colorectal adenocarcinoma. *Eur J Cancer* **27**: 1646–1649
Sun XF, Carsten JM, Zhang H, Stal O, Wingern S, Hatschek T and Nordenskjold B (1992) Prognostic significance of cytoplasmic p53 oncoprotein in colorectal adenocarcinoma. *Lancet* **340**: 1369–1373
Sun XF, Carstensen JM, Stal O, Zhang H and Nordenskjold B (1995) *c-erb* B2 oncoprotein in relation to DNA ploidy and prognosis in colorectal adenocarcenoma. *APMIS* **103**: 309–315
Sun XF, Carstensen JM, Stal O, Zhang H and Nordenskjold B (1996) Proliferating cell nuclear antigen (PCNA) in relation to ras, *c-erb*B-2, p53, clinico-pathological variables and prognosis in colorectal adenocarcinoma. *Int J Cancer* **69**: 5–8
Takahashi Y, Tucher SL, Kitadai Y, Koura AN, Bucana CD, Cleary KR and Ellis LM (1997) Vessel counts and expression of vascular endothelial growth factor as prognostic factors in node-negative colon cancer. *Arch Surg* **132**: 541–546
Takebayashi Y, Akiyama S-I, Akiba S, Yamada K, Miyadera K, Sumizawa T, Yamada Y, Murata F and Aikou T (1996) Clinicopathologic and prognostic significance of an angiogenic factor, thymidine phosphorylase, in human colorectal carcinoma. *J Natl Cancer Inst* **88**: 1110–1117
Wadler S, Bajaj R, Neuberg D, Agarwal V, Haynes H and Benson ALB (1997) Prognostic implications of c-Ki-ras2 mutations in patients with advanced colorectal cancer treated with 5-fluorouracil and interferon: A study of the Eastern Cooperative Oncology Group (EST 2292). *Cancer J Scientific Am* **3**: 284–289
Witzig TE, Lopfinzi CL, Gonchoroff NJ, Reiman HM, Cha SS, Wieand HS, Katzmann JA, Paulsen JK and Moertel CG (1991) DNA ploidy and cell kinetic measurements as predictors of reoccurrence and survival in stages B2 and C colorectal adenocarcinoma. *Cancer* **68**: 879–888
Wolmark N, Wieand HS, Rockette HE, Fisher B, Glass A, Lawrence W, Lerner H, Cruz AB, Volk H, Shibata H, Evans J and Prager D (1983) The prognostic significance of tumour location and bowel obstruction in Dukes' B and C colorectal cancer. *Ann Surg* **198**: 743–750
Wynford-Thomas D (1992) p53 in tumour pathology: can we trust immunohistochemistry? *J Pathol* **166**: 329–330
Yamaguchi A, Nakagawara G, Kurosaka Y, Nishimura G, Yonemura Y and Miyazaki I (1993) P53 immunoreaction in endoscopic biopsy specimens of colorectal cancer, and its prognostic significance. *Br J Cancer* **68**: 399–402
Yamaguchi A, Urano T, Goi T, Saito M, Takeuchi K, Hirose K, Nakagawara G, Shiku H and Furukawa K (1996) Expression of a CD44 variant containing exons 8 to 10 is a useful independent factor for the prediction of prognosis in colorectal cancer patients. *J Clin Oncol* **14**: 1122–1127
Younes M, Fernandez L and Lechago J (1996) Transforming growth factor alpha (TGF-α) expression in biopsies of colorectal carcinoma is a significant prognostic indicator. *Anticancer Res* **16**: 1999–2004
Zeng ZS, Sarkis AS, Zhang ZF, Klimstra DS, Charytonowicz E, Guillem JG, Cordon-Cardo C and Cohen AM (1994) P53 nuclear overexpression – an independent predictor of survival in lymph node positive colorectal cancer patients. *J Clin Oncol* **12**: 2043–2050
Zeng ZS, Huang Y, Cohen AM and Guillem JG (1996) Prediction of colorectal cancer relapse and survival via tissue RNA levels of matrix metalloproteinase-9. *J Clin Oncol* **14**: 3133–3140

© Cancer Research Campaign 1999
